# The comparison of acute toxicities associated with craniospinal irradiation between photon beam therapy and proton beam therapy in children with brain tumors

**DOI:** 10.1002/cam4.4553

**Published:** 2022-02-09

**Authors:** Suguru Uemura, Yusuke Demizu, Daiichiro Hasegawa, Tomoko Fujikawa, Shotaro Inoue, Akihiro Nishimura, Ryunosuke Tojyo, Sayaka Nakamura, Aiko Kozaki, Atsuro Saito, Kenji Kishimoto, Toshiaki Ishida, Takeshi Mori, Jyunji Koyama, Atsufumi Kawamura, Yoshinobu Akasaka, Makiko Yoshida, Nobuyoshi Fukumitsu, Toshinori Soejima, Yoshiyuki Kosaka

**Affiliations:** ^1^ Department of Hematology and Oncology Kobe Children’s Hospital Kobe Japan; ^2^ Department of Radiation Oncology Kobe Proton Center Kobe Japan; ^3^ Department of Neurosurgery Kobe Children’s Hospital Kobe Japan; ^4^ Department of Radiology Kobe Children’s Hospital Kobe Japan; ^5^ Department of Pathology Kobe Children’s Hospital Kobe Japan

**Keywords:** brain tumor, craniospinal irradiation, pediatrics, photon beam therapy, proton beam therapy

## Abstract

**Introduction:**

This study aimed to evaluate acute toxicities associated with irradiation between the X‐CSI (photon beam craniospinal irradiation) and P‐CSI (proton beam craniospinal irradiation) groups in children with brain tumors.

**Methods:**

Sixty‐two consecutive patients who received initial craniospinal irradiation (CSI) for brain tumors in our center between January 1, 2011 and May 31, 2021, were included in the study. Acute toxicities were retrospectively evaluated during CSI using Common Terminology Criteria for Adverse Events version 5.0. Maximum grades of fatigue, headache, insomnia, nausea, vomiting, dermatitis, constipation, abdominal pain, oropharyngeal mucositis, and hematological toxicities were evaluated.

**Results:**

Thirty‐six patients received X‐CSI, and 26 patients received P‐CSI. The median dose of CSI was 18.0 Gy in the X‐CSI group and 23.4 Gy (relative biological effectiveness) in the P‐CSI group (*p* < 0.001). The P‐CSI group had a lower incidence of more than grade 2 nausea (11.5% vs. 69.4%, *p* = 0.008) and vomiting (7.7% vs. 38.8%, *p* < 0.001), compared with the X‐CSI group. Multivariate logistic regression analysis with adjustments for potential confounding factors of doses of CSI showed that proton radiation therapy was associated with a marked reduced risk of more than grade 2 nausea and vomiting during CSI (adjusted odds ratio, 0.050; 95% confidential interval, 0.011–0.24; *p* < 0.001).

**Conclusion:**

The present study suggests that P‐CSI reduces the acute gastrointestinal toxicities associated with irradiation.

## Graphical abstract

Our main findings were that proton radiation therapy was associated with a marked reduced risk of more than grade 2 nausea and vomiting during craniospinal irradiation (CSI). This result suggests that proton beam CSI reduces acute gastrointestinal toxicities associated with irradiation.

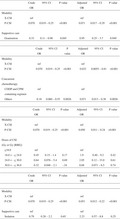

## 1 INTRODUCTION

Radiation therapy (RT) has an important role in the multidisciplinary treatment of brain tumor in children. Craniospinal irradiation (CSI) is usually indicated for medulloblastoma (MBL), embryonal tumor with multilayered rosettes, and some germ cell tumors, ependymoma, and atypical teratoid rhabdoid tumors.^1^, ^2^, ^3^, ^4^, ^5^ CSI has contributed to the improvement of survival of these diseases. However, CSI effects on significant acute and late side effects.^6^, ^7^

To reduce the dose to normal tissue at risk keeping the optimal dose for the target coverage, proton radiation therapy (PRT) has used as an alternative technique. Photons emit maximal energy near the body surface; this energy gradually decreases at deeper points in the body. Hence, photon radiation therapy (XRT) is closely related with various acute and late adverse events.^7^, ^8^, ^9^ In contrast, protons deposit a relatively low dose near the body surface and emit maximum energy just before they stop inside the body (the Bragg peak effect).^10^, ^11^ The Bragg peak effect may be spread according to the location and size of the tumor, making it possible to deliver high‐dose radiation to the tumor, while limiting the dose delivered to the organs at risk. The biological effects of protons are almost identical to those of photons (relative biological effectiveness [RBE], 1.1).^12^

PRT can reduce damage to the normal tissue and offers an obvious advantage in reducing dose of organ at risk compared with XRT.^13^, ^14^, ^15^, ^16^, ^17^ PRT reduces the radiation‐induced acute and late morbidities for patients with childhood cancers.^8^, ^18^, ^19^, ^20^, ^21^, ^22^

Although the number of patients receiving PRT is increased worldwide, there is limited data focused on the comparison of acute toxicities associated with CSI between XRT and PRT in children with brain tumors.^23^, ^24^

This retrospective cohort study aimed to evaluate acute toxicities associated with irradiation between the X‐CSI (photon beam craniospinal irradiation) and P‐CSI (proton beam craniospinal irradiation) groups in children with brain tumors.

## 2 MATERIALS AND METHODS

### 2.1 Study population

A total of 63 consecutive patients who received initial CSI for brain tumors in Kobe Children's Hospital and Kobe Proton Center between January 1, 2011 and May 31, 2021 were included in the study. The patients treated between January 1, 2011 and September 30, 2019, received CSI as XRT, while the patients treated between February 1, 2019 and May 31, 2021, received CSI as PBT. One patient was excluded because of discontinuation of CSI due to disease progression. Finally, we retrospectively analyzed data of 62 patients from the clinical records. Baseline patients' characteristics were obtained from their medical records.

This study was approved by the ethics review committees of Kobe Children's Hospital and Kobe Proton Center.

### 2.2 Evaluation of acute toxicities

Acute toxicities during CSI were retrospectively evaluated using Common Terminology Criteria for Adverse Events version 5.0. Maximum grade of fatigue, headache, insomnia, nausea, vomiting, dermatitis, constipation, abdominal pain, oropharyngeal mucositis, and hematological toxicities, including white blood cell (WBC) count decreased, anemia, and platelet count decreased, were evaluated. The observational period was from the first day of CSI to the next day after completion of CSI.

### 2.3 Concurrent chemotherapy

To evaluate the association of concurrent chemotherapy with acute toxicities during CSI, we divided the regimens of concurrent chemotherapies into three groups due to the heterogeneity of the regimens. The first group consisted of cisplatin (CDDP) and cyclophosphamide (CPM) containing regimen.^25^, ^26^, ^27^ The regimens of this group were as follows: (I) vincristine (VCR) (1.5 mg/m^2^ on day 1), CDDP (90 mg/m^2^ on day 2), and CPM (1000 mg/m^2^ on days 1, 3, and 5 or on days 1–3), (II) CDDP (90 mg/m^2^ on day 2), CPM (1000 mg/m^2^ on day 1), and etoposide (ETP) (100 mg/m^2^ on days 1–5), and (III) VCR (1.5 mg /m^2^ on day 1), CDDP (90 mg/m^2^ on day 2), CPM (1200 mg/m^2^ on day 1), and pirarubicin (40 mg/m^2^ on day 1). The second group included weekly VCR: VCR at a dose of 1.5 mg/m^2^ weekly until the completion of RT. The third group consisted of other regimens: (I) oral ETP (60 mg/m^2^ daily), (II) temozolomide (150 mg/m^2^ on days 1–5) and irinotecan (50 mg/m^2^ on days 1–5), and (III) topotecan (0.75 mg/m^2^ on days 1–5) and CPM (250 mg/m^2^ on days 1–5).

Methotrexate (MTX)‐containing intrathecal chemotherapy consisted of MTX and dexamethasone (DEX). The dose of these drugs were defined according to age as follows: MTX (age <11 months, 6 mg; 1 year, 8 mg; 2 years, 10 mg; >3 years, 12 mg) and DEX (age <11 months, 4 mg; 1 year, 5 mg; 2 years, 6 mg; >3 years, 8 mg).

### 2.4 Supportive care during CSI

All patients, other than one patient, received CSI in Kobe Children's Hospital. All patients received anti‐pneumocystis prophylaxis and antifungal therapy using oral sulfamethoxazole/trimethoprim. When the neutrophil count was <500/μL, granulocyte‐colony stimulating factor, and fluconazole were administered.

All patients who received concurrent chemotherapy received granisetron (40 μg/kg dose) from day 1 to the last day of each regimen. All patients who received CDDP as concurrent chemotherapy received oral aprepitant or fosaprepitant.

During CSI, granisetron, osmotic diuretics, and sodium alginate were administered at the physician's discretion.

### 2.5 CSI technique

The radiation treatments were planned using a computed tomography (CT)‐based three‐dimensional treatment planning system. Each patient was immobilized using a custom‐made thermoplastic cast in the supine position; then, CT was performed. The target volumes and organs at risk were delineated on the CT images basically, and the CT magnetic resonance imaging fusion images were also used, if necessary. The clinical target volume (CTV) included the entire cranial and spinal meninges. The planning target volume was defined as the CTV plus a setup margin (XRT, 5 mm for cranial CTV and 10 mm for spinal CTV; PRT, 3 mm for cranial CTV and 6 mm for spinal CTV), and the vertebral bodies were included if the patient was aged <10 years to prevent scoliosis. The dose constraints were defined only for CTV and the lens: CTV, *D*_min_ (minimum dose) ≥90% of the prescribed dose (PD), and *D*_max_ (maximum dose) <105% of PD; lens, *D*_max_ <10 Gy (RBE). Table 1 shows the comparison of radiation technique between XRT and PRT. The reported dose of PRT was calculated by multiplying the physical dose by the RBE of the protons (1.1).

**TABLE 1:** Comparison of radiation technique

	XRT (*N* = 36)	PRT (*N* = 26)
Technique	3D‐CRT	BRD (*N* = 9) SCN (*N* = 17)
Treatment planning system	XiO (Elekta, Stockholm, Sweden)	RayStation (RaySearch Laboratories, Stockholm, Sweden)
Fractionation time (minutes)	20	70 (BRD) 60 (SCN)
Frequency of portal images	First time only	Daily

Abbreviations: 3D‐CRT, three‐dimensional conformal radiation therapy; BRD, broad beam; PRT, proton radiation therapy; SCN, scanning; XRT, photon radiation therapy.

### 2.6 Statistical analysis

In the present study, the primary outcome was to evaluate acute toxicities associated with irradiation between the X‐CSI and P‐CSI groups. Chi‐square test or Fisher's exact test was used to compare categorical variables in patients' characteristics between the X‐CSI and P‐CSI groups. Unpaired *t*‐test or Mann–Whitney *U* test was also used to compare continuous variables in patients' characteristics between the X‐CSI and P‐CSI groups. To compare the maximum grade of acute toxicities associated with irradiation, chi‐square test or Fisher's exact test was used. The logistic regression model was used to calculate the adjusted odds ratio (OR) with 95% confidence interval (CI) for the development of more than grade 2 nausea and vomiting associated with the modality of CSI. The multivariate logistic regression model was performed with adjustments for the potential confounding factors of the use of granisetron during CSI, regimens of concurrent chemotherapy, need sedation during CSI, and doses of CSI. For all models, the number of examined covariates was determined by the number of outcome events with 10 events required for one covariate. A *p* < 0.05 was considered statistically significant. Statistical analyses were performed using EZR (Saitama Medical Center, Jichi Medical University), a modified version of R commander (The R Foundation for Statistical Computing).^28^

## 3 RESULTS

### 3.1 Patients and characteristics

Table 2 showed the clinical characteristics of the patients. The median age at CSI was 6.8 years (0.5–18.1) in the X‐CSI group and 7.8 years (1.7–16.5) in the P‐CSI group (*p* = 0.22). There was no significant difference in female/male ratio between the two groups (*p* = 0.30). In both groups, MBL accounted for the most common as the primary disease (55.6% in the X‐CSI group vs. 73.1% in the P‐CSI group).

**TABLE 2:** Clinical characteristics of the present cohort

Characteristic	X‐CSI group (*N* = 36)	P‐CSI group (*N* = 26)	*p* value
Patients			
Median age at CSI (year)	6.8	7.8	0.22
(range)	0.5–18.1	1.7–16.5	
Gender			0.30
Female	18 (50.0%)	17 (65.4%)	
Male	18 (50.0%)	9 (34.6%)	
Diseases			
Primary diagnosis			0.22
Medulloblastoma	20 (55.6%)	19 (73.1%)	
ETMR	3 (8.3%)	1 (3.8%)	
Germ cell tumor	5 (13.9%)	2 (7.7%)	
AT/RT	5 (13.9%)	0 (0.0%)	
Others	3 (8.3%)	3 (11.5%)	
Primary site			0.63
Supratentorial	13 (36.1%)	7 (26.9%)	
Infratentorial	23 (63.9%)	19 (73.1%)	
Radiation			
Median CSI dose (Gy or Gy [RBE])	18	23.4	<0.001
≤18.0	20 (55.6%)	3 (11.5%)	
<18.0 to ≤24.0	11 (30.6%)	18 (69.3%)	
<24.0 to ≤30.0	2 (5.5%)	2 (7.7%)	
<30.0 to ≤36.0	3 (8.3%)	3 (11.5%)	
Dose per fraction (Gy or Gy [RBE])			<0.001
1.5	25 (69.4%)	0 (0.0%)	
1.8	11 (30.6%)	26 (100%)	
Subsequent RT	35 (97.2%)	25 (96.2%)	1.00
Focal	32 (91.4%)	24 (96.0%)	
Whole brain	3 (8.6%)	1 (4.0%)	
Vertebral body sparing	11 (30.6%)	10 (38.5%)	0.59
Chemotherapy			
Concurrent chemotherapy	28 (77.8%)	24 (92.3%)	0.17
CDDP + CPM containing	21 (75.0%)	15 (62.5%)	0.17
Weekly VCR	3 (10.7%)	6 (25.0%)	
Others	4 (14.3%)	3 (12.5%)	
Intrathecal MTX	18 (50.0%)	15 (57.7%)	0.44
Received chemotherapy prior to CSI	26 (72.2%)	18 (69.2%)	1.00
Supportive care			
Granisetron	16 (44.4%)	23 (88.5%)	0.008
Osmotic diuretics	8 (22.2%)	2 (7.7%)	0.17
Sodium alginate	10 (27.8%)	1 (2.8%)	0.018
Need sedation during CSI	21 (80.8%)	18 (50.0%)	0.02

Abbreviations: AT/RT, atypical teratoid rhabdoid tumors; CDDP, cisplatin; CPM, cyclophosphamide; CSI, craniospinal irradiation; ETMR, embryonal tumor with multilayered rosettes; MTX, methotrexate; P‐CSI, proton beam craniospinal irradiation; RBE, relative biological effectiveness; RT, radiation therapy; VCR, vincristine; X‐CSI, photon beam craniospinal irradiation.

The median dose of CSI was 18 Gy (12.0–36.0) in the X‐CSI group and 23.4 Gy (RBE) (18.0–36.0) in the P‐CSI group (*p* < 0.001). Eleven (30.5%) patients in the X‐CSI group and all patients in the P‐CSI group received the radiation with daily fractional dose of 1.8 Gy (RBE) (*p* < 0.001). There was no significant difference between the X‐CSI and P‐CSI groups in the number of patients who received subsequent RT (*p* = 1.00). Thirty‐five patients (97.2%) in the X‐CSI group and 25 patients (96.2%) in the P‐CSI group received CSI, followed by focal or whole brain irradiation.

In both groups, CDDP and CPM containing regimen was the most common concurrent chemotherapy (65.0% in the X‐CSI group vs. 75.0% in the P‐CSI group). There was no significant difference in the use of intrathecal MTX during CSI between the X‐CSI and P‐CSI groups (50.0% vs. 57.5%, *p* = 0.44).

### 3.2 Acute toxicity profiles during CSI

Acute toxicity profiles during CSI are summarized in Table 3. In the X‐CSI group, 11 patients (30.5%) developed grade 2 nausea, whereas two patients (7.7%) experienced grade 2 nausea in the P‐CSI group. Three patients (8.3%) had grade 3 nausea in the X‐CSI group, while there were no patients with grade 3 nausea in the P‐SCI group. In the X‐CSI group, 16 patients (44.4%) had grade 2 vomiting, whereas three patients (11.5%) experienced grade 2 vomiting in the P‐CSI group. Nine patients (25.0%) experienced grade 3 vomiting in the X‐CSI group, while there were no patients who experienced grade 3 vomiting in the P‐CSI group (Table 3). There was a significant difference in the incidence of more than grade 2 nausea and vomiting between the X‐CSI and P‐CSI groups (38.8% vs. 7.7%, *p* = 0.008, and 69.4% vs. 11.5%, *p* < 0.001, respectively).

**TABLE 3:** Maximum grade of acute toxicity during CSI

Characteristic	X‐CSI group (*N* = 36)	P‐CSI group (*N* = 26)	*p* value
Fatigue			0.053
0	5	11	
1	19	10	
2	11	4	
3	1	1	
Headache			0.241
0	24	17	
1	4	7	
2	7	2	
3	1	0	
Insomnia			0.566
0	30	22	
1	5	2	
2	1	2	
Nausea			0.020
0	7	12	
1	15	12	
2	11	2	
3	3	0	
Vomiting			<0.001
0	4	7	
1	7	16	
2	16	3	
3	9	0	
Dermatitis			0.54
0	15	8	
1	18	17	
2	3	1	
Constipation			0.341
0	18	8	
1	7	6	
2	11	12	
Abdominal pain			0.214
0	25	19	
1	9	3	
2	2	4	
Oropharyngeal pain			0.903
0	17	12	
1	4	3	
2	10	9	
3	5	2	

Abbreviations: P‐CSI, proton beam craniospinal irradiation; X‐CSI, photon beam craniospinal irradiation.

Differences in the maximum grade of fatigue, headache, insomnia, constipation, abdominal pain, and oropharyngeal pain were not significant between the X‐CSI and P‐CSI groups (Table 3). Acute hematological toxicity profiles are shown in Table 4. In WBC count decreased, anemia, and platelet count decreased, there were no significant differences between the X‐CSI and P‐CSI groups. There were also no significant differences in neutropenia, episodes of febrile neutropenia, and needs of transfusions between the X‐CSI and P‐CSI groups. We investigated acute hematological toxicity profiles among patients who received irradiation with vertebral body sparing. Eleven patients (30.6%) in the X‐CSI group and 10 patients (38.5%) in the P‐CSI group received vertebral body sparing (*p* = 0.59). In patients who received irradiation with vertebral body sparing, there were no significant differences in WBC count decreased, anemia, and platelet count decreased between the X‐CSI and P‐CSI groups. There were also no significant differences in neutropenia, episodes of febrile neutropenia, and needs of transfusions between the X‐CSI and P‐CSI groups.

**TABLE 4:** Maximum grade of acute hematological toxicity and profiles during CSI

Characteristic	X‐CSI group (*N* = 36)	P‐CSI group (*N* = 26)	*p* value
WBC decreased			0.091
1	1	0	
2	2	0	
3	3	7	
4	30	19	
Anemia			0.255
0	1	0	
1	1	0	
2	4	2	
3	7	11	
4	23	13	
Platelet count decreased			0.504
0	3	1	
1	5	3	
2	3	6	
3	11	5	
4	14	11	
Neutropenia (<500/μL)	31	18	0.13
Febrile neutropenia	14	8	0.60
Transfusion	14	10	1.00

Abbreviations: X‐CSI, photon beam craniospinal irradiation; P‐CSI, proton beam craniospinal irradiation; WBC, white blood cell.

To identify the risk factor of more than grade 2 nausea and vomiting, multivariate logistic regression analysis was performed with adjustments for the potential confounding factors of use of granisetron during CSI, sedation during CSI, regimens of concurrent chemotherapy, and doses of CSI. Table 5 shows the adjusted OR of more than grade 2 nausea and vomiting during CSI. P‐CSI had an association with a marked reduction in risk of more than grade 2 nausea and vomiting during CSI. Granisetron had no association with a reduced risk of more than grade 2 nausea and vomiting (adjusted OR, 0.95; 95% CI, 0.251–3.65). Sedation during CSI also had no association with a reduced risk of more than grade 2 nausea and vomiting (adjusted OR, 2.23; 95% CI, 0.57–8.8). CDDP and CPM containing regimen was associated with a significant increase of risk of more than grade 2 nausea and vomiting. Doses of CSI had no association with an increased risk of more than grade 2 nausea and vomiting.

**TABLE 5:** Multivariate logistic regression models for more than grade 2 nausea and vomiting

	Crude OR	95% CI	*p* value	Adjusted OR	95% CI	*p* value
Modality						
X‐CSI	Ref			Ref		
P‐CSI	0.070	0.019–0.25	<0.001	0.071	0.017–0.29	<0.001
Supportive care						
Granisetron	0.33	0.11–0.98	0.045	0.95	0.25–3.7	0.949
Modality						
X‐CSI	Ref			Ref		
P‐CSI	0.070	0.019–0.25	<0.001	0.032	0.0055–0.81	<0.001
Concurrent chemotherapy						
CDDP and CPM containing regimen	Ref			Ref		
Others	0.18	0.060–0.55	0.0026	0.071	0.013–0.38	0.0026
Modality						
X‐CSI	Ref			Ref		
P‐CSI	0.070	0.019–0.25	<0.001	0.050	0.011–0.24	<0.001
Doses of CSI (Gy or Gy [RBE])						
≤18.0	Ref			Ref		
<18.0 to ≤24.0	0.45	0.15–1.4	0.17	1.9	0.40–9.2	0.42
<24.0 to ≤30.0	0.64	0.076–5.4	0.69	2.05	0.12–33.0	0.61
<30.0 to ≤36.0	0.32	0.048–2.1	−0.24	0.68	0.071–6.5	0.74
Modality						
X‐CSI	Ref			Ref		
P‐CSI	0.070	0.019–0.25	<0.001	0.051	0.012–0.22	<0.001
Supportive care	Ref			Ref		
Sedation	0.79	0.28–2.2	0.65	2.23	0.57–8.8	0.25

Abbreviations: CDDP, cisplatin; CI, confidence interval; CPM, cyclophosphamide; CSI, craniospinal irradiation; OR, odds ratio; RBE, relative biological effectiveness.

## 4 DISCUSSION

Herein, we compared the acute toxicity profiles between the X‐CSI and P‐CSI groups. In this study, we revealed that the incidence rates of more than grade 2 nausea and vomiting in the P‐CSI group were lower than in the X‐CSI group, although P‐CSI group had higher doses of CSI than the X‐CSI group. Nausea and vomiting are common side effects of RT.^29^ Nausea and vomiting often reduce patients' quality of life (QOL) and nutrition status.^29^ Therefore, the result of the present study is considerably interesting to improve patients’ QOL during CSI. Differences in the maximum grade of other gastrointestinal toxicities such as constipation, abdominal pain, and oropharyngeal pain were not significant between the X‐CSI and P‐CSI groups. If the dose of CSI was same in the two groups, the incidence of abdominal pain in X‐CSI group might be significantly higher because of the increase of the dose delivered to the gastrointestinal tract.

A previous study in adults showed that patients who received P‐CSI had significantly lower rates of acute gastrointestinal toxicities, including weight loss, nausea, vomiting, and anorexia compared with patients receiving X‐CSI.^30^ In the present study, the incidence rates of nausea and vomiting were lower in the P‐CSI group than in the X‐CSI group. This result can be attributed to the significant reduction in doses to the esophagus, stomach, and bowel.^30^

In our center, patients who needed sedation during irradiation stopped eating 6 h before irradiation and drinking clear fluids 2 h before irradiation. For this reason, all patients who needed sedation could not eat during CSI. Sedation during CSI reduced the amounts of meals obviously. Hence, the incidence rates of anorexia and weight loss were not examined in the present study. In general, P‐CSI takes longer times to perform than X‐CSI. The number of patients who needed sedation during CSI was higher in the P‐CSI group than in the X‐CSI group, (*p* = 0.02, Table 2). However, sedation had no association with a reduced risk of more than grade 2 nausea and vomiting (Table 5).

A previous study in adults showed that patients who received P‐CSI had significantly lower rates of acute hematological toxicities than patients receiving X‐CSI because P‐CSI can spare the bone marrow within the spinal column.^30^ On the other hand, in younger cases, their vertebral bodies receive an approximately uniform dose of radiation to prevent scoliosis as a late effect. Therefore, in older cases, P‐CSI also reduces the incidence and severity of hematological toxicities.^23^, ^24^

However, in our study, there were no significant differences in WBC count decreased, anemia, and platelet count decreased between the X‐CSI and P‐CSI groups. This difference could be explained by the intensity of concurrent chemotherapy. In our study, 21 patients (58.3%) in the X‐CSI group, 15 patients (57.7%) in the P‐CSI group received CDDP and CPM containing regimen. This regimen consisted of CDDP (90 mg/m^2^ for 1 day) and CPM (1000–1200 mg/m^2^ for 1 day or 3 days) and induces greater myelosuppression than other regimens, such as weekly VCR.^25^, ^26^, ^27^, ^31^, ^32^ A small number of patients receiving irradiation with vertebral body sparing in the P‐CSI group also failed to indicate the reduction of acute hematological toxicity compared with those in the X‐CSI group. Further studies with uniform concurrent chemotherapy are required to evaluate whether P‐CSI reduces the acute hematological toxicities associated with irradiation.

Generally, the incidences of acute toxicity associated with irradiation are expected to be higher if the CSI doses are higher. However, the present study did not show the association of more than grade 2 nausea and vomiting with the CSI doses (Table 5). This difference could be explained by the small number of patients receiving CSI doses of 30.0–36.0 Gy (Table 2). In these patients, only one patient received CDDP and CPM containing regimen. Therefore, the CSI dose was not associated with an increased risk of more than grade 2 nausea and vomiting in the present study.

The present study has several limitations. First, this study involved a retrospective analysis in a single center including a small number of patients. Second, the treatment period was different between the P‐CSI and X‐CSI groups. Supportive care during CSI depended on the physician's choice and might affect the incidences of nausea and vomiting during CSI. However, granisetron had no association with a reduced risk of more than grade 2 nausea and vomiting (Table 5). A prospective study investigating the acute toxicities of P‐CSI versus X‐CSI would be difficult to perform with large numbers of patients. Lastly, the concurrent chemotherapy regimens were heterogeneous. The benefits of PRT in reducing acute toxicity in CSI could not be fully explained. Therefore, further studies in larger cohorts are required to evaluate the acute toxicity profiles during CSI.

In conclusion, in the present study, although the P‐CSI group received higher CSI doses than the X‐CSI group, the incidence rates of more than grade 2 nausea and vomiting in the P‐CSI group were lower than in the X‐CSI group. The present study suggests that P‐CSI reduces the incidence of acute gastrointestinal toxicities associated with irradiation.

## CONFLICT OF INTEREST

The authors report no conflict of interest.

## ETHICAL APPROVAL

The present study was approved by the Institutional Review Board of Kobe Children's Hospital (R03‐57). Because of the nature of the retrospective analysis, informed consent, or assent was waived and opt‐out system was presented.

## AUTHOR CONTRIBUTIONS

Suguru Uemura: Treatment of patients, conceptualization and study design, data collection and interpretation, writing‐original draft, and writing‐review and editing. Yusuke Demizu: Study design and methodology, data analysis and data interpretation, and writing of the manuscript. Daiichiro Hasegawa: Conceptualization, study design, methodology, data analysis, data interpretation, critically revised article. Tomoko Fujikawa: Treatment of patients and data extraction. Shotaro Inoue: Treatment of patients and data extraction. Akihiro Nishimura: Treatment of patients and data extraction. Ryunosuke Tojyo: Treatment of patients and data extraction. Sayaka Nakamura: Treatment of patients and data acquisition. Aiko Kozaki: Treatment of patients and data acquisition. Atsuro Saito: Treatment of patients and data extraction. Kenji Kishimoto: Treatment of patients, data extraction, analysis, and interpretation of the data. Toshiaki Ishida: Treatment of patients and data extraction. Takeshi Mori: Treatment of patients and data extraction. Jyunji Koyama: Treatment of patients, data extraction, and analysis and interpretation of the data. Atsufumi Kawamura: Treatment of patients, data extraction, and analysis and interpretation of the data. Yoshinobu Akasaka: Treatment of patients, data extraction, analysis, and interpretation of the data. Makiko Yoshida: Treatment of patients, data, analysis, interpretation of the data, and critically revised article. Nobuyoshi Fukumitsu: Treatment of patients, data acquisition, formal data analysis, and data interpretation. Toshinori Soejima: formal data analysis and data interpretation, and writing of the manuscript. Yoshiyuki Kosaka: Conceptualization, study design, methodology, formal data analysis, and data interpretation. All authors have approved the final manuscript.

## Data Availability

The data that support the findings of this study are available from the corresponding author, upon reasonable request.
